# Supplementary material for: Infrared spectroscopic laser scanning confocal microscopy for whole-slide chemical imaging
Source: Nat Commun. 2023 Aug 25;14:5215. doi: 10.1038/s41467-023-40740-w (PMC10457288; doi:10.1038/s41467-023-40740-w)
Supplement: Supplementary file 1 — Supplementary Information [file 41467_2023_40740_MOESM1_ESM.pdf]

Supplementary Information

Infrared spectroscopic laser scanning confocal microscopy for whole-slide chemical imaging

Kevin Yeh[1,2], Ishaan Sharma[1], Kianoush Falahkheirkhah[1,3], Matthew P. Confer[1], Andres C. Orr[1], Yen-Ting Liu[1,4], Yamuna Phal[1,4], Ruo-Jing Ho[1,2], Manu Mehta[2], Ankita Bhargava[5], Wenyan Mei[6,7,8], Georgina Cheng[8,9], John C. Cheville[10], and Rohit Bhargava*[,1,2,3,4,8,11,12]

[1]Beckman Institute for Advanced Science and Technology, University of Illinois at Urbana-Champaign, Urbana, IL 61801, USA. [2]Department of Bioengineering, University of Illinois at Urbana-Champaign, IL 61801, USA. [3]Department of Chemical and Biomolecular Engineering, University of Illinois at Urbana-Champaign, Urbana, IL 61801, USA. [4]Department of Electrical and Computer Engineering, University of Illinois at Urbana-Champaign, Urbana, IL 61801, USA. [5]University of Illinois Laboratory High School, Urbana, IL 61801, USA. [6]Department of Comparative Biosciences, College of Veterinary Medicine, University of Illinois at Urbana-Champaign, Urbana, IL 61802, USA. [7]Carl R. Woese Institute for Genomic Biology, University of Illinois at Urbana-Champaign, Urbana, IL 61801, USA. [8]Cancer Center at Illinois, University of Illinois at Urbana-Champaign, Urbana, IL 61801, USA. [9]Carle Health, Urbana, IL 61801, USA. [10]Department of Laboratory Medicine and Pathology, College of Medicine and Science, Mayo Clinic, Rochester, MN 55905, USA. [11]Department of Chemistry, University of Illinois at Urbana-Champaign, Urbana, IL 61801, USA. [12]Department of Mechanical Science and Engineering, University of Illinois at Urbana-Champaign, Urbana, IL 61801, USA.

Corresponding author. Correspondence and requests for materials should be addressed to R.B. *E-mail: rxb@illinois.edu. Tel: (217) 265-6596. Address: 4265 Beckman Institute for Advanced Science and Technology, University of Illinois at Urbana-Champaign, 405 N Mathews Ave, Urbana, IL 61801.

List of Supplementary Tables and Figures

Table S1: Table of as-designed specifications of IR-LSM compared to other select IR imaging systems.

Table S2: Table of refractive indices used for lens optimizations.

Table S3: Methods of evaluating system spectral noise, spatial noise, and respective SNR for fig. 1c(i-iv).

Figure S1: Summary of aberration corrected optical design and performance.

Figure S2: Map of the material library used for lens optimizations.

Figure S3-6: Lens surface parameters of all custom optical designs.

Figure S7: Cross-sections of lens housings.

Figure S8: System control schematic of IR-LSM.

Figure S9: Measured achromatic performance of the optics and software autofocus algorithm.

General Information

This supplementary information contains a selection of reports listing engineering specifications and design performance targets for IR-LSM intended as an aid for expert readers to design similar mid-IR spectroscopic instruments in-house. As commercial off the shelf components are not available, and similar IR components (originally designed for surveillance or laser drilling and welding) will have subpar properties for microscopy while also being obscured in proprietary information, we have gathered and summarized key data in tabulated form. Additional information is available from the corresponding author upon request. We also describe objective methods for system evaluation using widely available standardized targets, should readers wish to themselves compare the performance of IR-LSM with the field. We begin this supplementary information with a comprehensive glossary of terms and abbreviations.

Glossary

*	Aspheric surfaces of the form: $z(r) = \frac{r^2}{R\left(1+\sqrt{1-(1+\kappa)\frac{r^2}{R^2}}\right)} + \alpha_4 r^4 + \alpha_6 r^6 + \cdots$ where displacement (z) of the surface along the optical axis at distance (r) from the axis is defined by the base radius of curvature (R), conic constant (κ), and n^{th} order aspheric coefficients (α_n).
∞	Infinity-corrected path of parallel light rays.
λ	Wavelength [µm].
λ_0	Design wavelength (within the tunable range of a narrowband QCL).
ν	Wavenumber [cm^{-1}].
σ	Standard deviation (stdev).
θ_{XY}	XY galvanometer (galvo) mirror scanner.
A	Absorbance unit [au].
A_I	Illumination aperture.
A_{RMS}	Absorbance RMS noise.
A_S	Aperture stop.
AFM	Atomic force microscopy.
AFS	Autofocus stabilization.
AI	Artificial intelligence.
AMTIR#	Series of IR glasses produced by Amorphous Materials Incorporated: (1) $Ge_{33}As_{12}Se_{55}$, (2) $As_{40}Se_{60}$.
AR	Anti-reflective.
AUC ROC	Area under the curve of a receiver operating curve.
BB	Broadband (λ ~ 2-12 µm).
BC	Beam combiner, here, a set of BS and M.
BS	Beam splitter.
BD	Beam dump.
CAD	Computer aided design.
CFS	Chromatic focal shift.
CI	Chemical imaging.
CVD	Chemical vapor deposition.
DAQ	Data acquisition system.
DF	Discrete frequency, alternatively DF infrared (DFIR).
EC	External cavity, relating to a specific QCL design capable of widely tunable narrowband emission.
FAST	Features from accelerated segment test, used for object tracking and stabilization.
FDA	U.S. Food and Drug Administration.
FFPE	Formalin-fixed, paraffin-embedded.
FM	Flip mirror (alternatively, a dichroic BS).
FOV	Field of view.
FPA	Focal-plane array (detector in IR cameras).
FPGA	Field-programmable gate array.
FT	Fourier transform.
G	Grating.
GASIR#	Series of IR glasses produced by Umicore: (1) $Ge_{22}As_{20}Se_{58}$.
H&E	Hematoxylin and eosin (stain) for histology.
IG#	Series of IR glasses produced by VITRON Spezialwerkstoffe GmbH: (2) $Ge_{33}As_{12}Se_{55}$, (6) $As_{40}Se_{60}$.
IOR	Index of refraction.
IR	Infrared (λ ~ 2-12 µm).
IRG#	Series of IR glasses produced by Schott AG: (22) $Ge_{33}As_{12}Se_{55}$, (23) $Ge_{30}Sb_{13}Se_{32}Te_{25}$, (24) $Ge_{10}As_{40}Se_{50}$, (25) $Ge_{28}Sb_{12}Se_{60}$, (26) $As_{40}Se_{60}$, (27) $As_{40}S_{60}$.
ISO	International Organization for Standardization.
KRS#	Series of IR transparent crystals "*Kristalle aus dem Schmelz-fluss*" developed by Carl Zeiss AG: (5) TlBr-TlI.
Low-e	IR-reflective low-emissivity glass coating.
lp	Line pair, unit for spatial frequency evaluations [lp/mm].
LSM	Laser scanning microscopy (by steering the beam whilst point-by-point detection by a single element sensor in conjunction with stage tiling).
LW	Long-wave infrared (λ ~ 8-15 µm).
M	Mirror.
Max-pool	Feature accumulation by down-sampling convolutional layers by maximum value, used in convolutional neural networks.
MCT	Mercury cadmium telluride (HgCdTe).
MEMS	Microelectromechanical systems.
MS-ZnS	Multispectral zinc sulfide, also known as Cleartran and clear-grade ZnS.
MTF	Modulation transfer function used to evaluate the spatial resolution limit of imaging systems.
MW	Mid-wave infrared (λ ~ 3-8 µm).
NA	Numerical aperture.
OAPM	Off-axis parabolic mirror.
OBJ	Microscopy objective, e.g. "10X / 0.4" represents "magnification / NA".
PV	Photovoltaic.
Px	Pixel, alternatively megapixel (MPx).
QCL	Quantum cascade laser.
RMS	Root mean square.
ROI	Region of interest.
S	Sample.
SaMD	Software as a medical device.
SC Ge	Single crystal germanium.
SL	Scan lens.
SNR	Signal to noise ratio.
SSM	Stage scanning microscopy (by translating the sample whilst point-by-point detection by a single element sensor).
TE	Thermoelectric.
TL	Tube lens.
U-Net	A convolutional neural network for semantic segmentation.
USAF 1951	Resolution test target defined by the U.S. Air Force MIL-STD-150A standard of 1951.
SU-8	An epoxy-based negative photoresist.
WFM	Widefield microscopy (by area detection with an arrayed sensor, such as an FPA, in conjunction with stage tiling).
WSI	Whole slide imaging.

Supplementary Tables and Figures

Specified Performance	(a) IR-LSM 10X / 0.4	(b) IR-LSM 20X / 0.8	(c) FT-IR 15X / 0.6	(d) DFIR SSM[21,22]	(e) DFIR SSM[21,22]	(f) DFIR WFM[14]
NA	0.4	0.8	0.62	0.56	0.85	0.56
Pixel size (µm)	2	1	1.1	2	1	2
FOV diameter† (µm)	2200	1100	141	N/A	N/A	256
Spatial res.†† (lp/mm)	79	128	70	114	168	45 – 77
Design wavelength (µm)	5.2-12	5.2-12	2-12	2.5	9.5	2.5
Anti-reflecting coating range (µm)	5.2-12	5.2-12	N/A	8-12	8-12	8-12
Corrected wavelengths	3	3	Full	1	1	1
Max. chromatic shift††† (µm)	3.3	1.7	0	57	23	57
RMS spot size, center-edge (µm)	0.3-0.8	1.7-1.8	1.0-1.3	2.7	9.3	14-27
Max. ray aberration (µm)	1.1	2.6	2.8	N/A	N/A	68
Max. optical path difference (waves)	0.02	0.06	0.107	N/A	N/A	1.7
Max. astigmatism (µm)	5.3	4.4	1.5	N/A	N/A	6
Max. distortion (%)	0.3	0.4	0	N/A	N/A	0.1

Table S1. As-designed specifications of (a-b) presented IR-LSM custom objective designs, compared to (c) an example FT-IR system, (d-e) a legacy custom point-by-point stage-scanning microscopy (SSM) DFIR system built in ref.[21,22], and (f) a legacy custom FPA-based widefield microscopy (WFM) DFIR system built in ref.[14], with both legacy systems fully reliant on commercial of the shelf IR components. Refer to fig. 1(d-e) for as-manufactured system performances. †The FOV for the IR-LSM 10X and 20X objectives were experimentally restricted to approximately half the design specification, which is typical for LSM systems without 3-mirror scanning designs to maintain optimal performance. ††All simulated results assume incoherent illumination, not coherent laser illumination; real-world DFIR systems will perform with partially coherent properties. The theoretical spatial resolution is calculated at λ = 6.25 µm. †††The theoretical maximum chromatic shift is calculated within λ = 5.2 to 12 µm.

λ [µm]	AMTIR1	GaAs	Ge	ZnSe	ZnS	MS-ZnS	GASIR1	BaF_2	AMTIR2	IRG26	IG2	IG6
5.0	2.511421	3.301047	4.016285	2.429527	2.246575	2.246616	2.507106	1.451024	2.779586	2.790898	2.510305	2.791030
5.4	2.510366	3.299098	4.014072	2.428083	2.243982	2.244120	2.506113	1.448391	2.778575	2.789711	2.509278	2.789835
5.8	2.509341	3.297266	4.012299	2.426603	2.241226	2.241463	2.505163	1.445543	2.777677	2.788621	2.508288	2.788738
6.2	2.508326	3.295504	4.010856	2.425071	2.238295	2.238630	2.504233	1.442476	2.776855	2.787596	2.507314	2.787706
6.6	2.507305	3.293778	4.009661	2.423476	2.235179	2.235608	2.503307	1.439184	2.776078	2.786608	2.506338	2.786711
7.0	2.506269	3.292062	4.008659	2.421809	2.231868	2.232388	2.502372	1.435662	2.775327	2.785641	2.505347	2.785737
7.4	2.505210	3.290338	4.007809	2.420063	2.228354	2.228958	2.501419	1.431905	2.774585	2.784682	2.504332	2.784770
7.8	2.504122	3.288590	4.007078	2.418232	2.224628	2.225309	2.500440	1.427908	2.773837	2.783719	2.503286	2.783799
8.2	2.503001	3.286806	4.006444	2.416310	2.220681	2.221430	2.499430	1.423664	2.773070	2.782746	2.502201	2.782818
8.6	2.501845	3.284975	4.005888	2.414294	2.216504	2.217311	2.498383	1.419167	2.772275	2.781755	2.501073	2.781819
9.0	2.500649	3.283088	4.005396	2.412179	2.212085	2.212942	2.497296	1.414409	2.771439	2.780742	2.499895	2.780797
9.4	2.499414	3.281135	4.004958	2.409962	2.207415	2.208312	2.496163	1.409384	2.770552	2.779703	2.498665	2.779750
9.8	2.498139	3.279110	4.004563	2.407640	2.202480	2.203408	2.494981	1.404083	2.769602	2.778635	2.497378	2.778672
10.2	2.496821	3.277002	4.004205	2.405207	2.197269	2.198218	2.493747	1.398498	2.768578	2.777534	2.496029	2.777561
10.6	2.495462	3.274804	4.003878	2.402662	2.191765	2.192728	2.492457	1.392619	2.767463	2.776398	2.494616	2.776416
11.0	2.494061	3.272505	4.003577	2.399999	2.185953	2.186923	2.491108	1.386437	2.766243	2.775226	2.493133	2.775232
11.4	2.492619	3.270095	4.003298	2.397217	2.179815	2.180788	2.489696	1.379940	2.764899	2.774014	2.491578	2.774009
11.8	2.491137	3.267563	4.003038	2.394311	2.173332	2.174306	2.488217	1.373118	2.763407	2.772761	2.489946	2.772745
12.2	2.489616	3.264898	4.002794	2.391277	2.166482	2.167457	2.486668	1.365958	2.761739	2.771466	2.488233	2.771438
12.6	2.488057	3.262086	4.002563	2.388111	2.159243	2.160223	2.485045	1.358447	2.759862	2.770126	2.486435	2.770087
13.0	2.486462	3.259113	4.002343	2.384809	2.151589	2.152580	2.483344	1.350570	2.757731	2.768741	2.484546	2.768689

Table S2. Refractive indices of allowed materials at each wavelength (λ) [µm] within the designed range. This is not a comprehensive list of IR compatible crystals and glasses, the list has been filtered for manufacturability, cost, and ease of procurement at the time of this study. These values can be derived from the Code V material database; however, verified material properties provided by the manufacturers should be used for accurate results. The chalcogenide glasses have the following compositions: AMTIR1 and IG2 ($Ge_{33}As_{12}Se_{55}$); GASIR1 ($Ge_{22}As_{20}Se_{58}$); AMTIR2, IRG26, and IG6 ($As_{40}Se_{60}$). Refractive indices of materials with identical compositions will differ by manufacturing process: AMTIR- is produced by Amorphous Materials Inc. (AMI), IG- is produced by VITRON Spezialwerkstoffe GmbH, GASIR- is produced by Umicore, and IRG- is produced by Schott AG. Multi-spectral ZnS is also known as Cleartran, an optically clear grade of ZnS.

a

100% Line

$\mathbf{A}$: absorbance image, $\mathbf{B}$: background region
$[x, y]$, $[\nu]$: spatial, spectral coordinates

Require: $[x, y] \in \mathbf{B}$
return $\mathbf{S}_{x,y} \leftarrow \mathbf{A}[x, y]\{\nu\}\ \forall\ \nu$

b

Spatial Noise

Require: $\{x, y\} \in \mathbf{B}$
for each $\nu \in \mathbf{A}$
  $\boldsymbol{\sigma}_\nu \leftarrow \mathrm{stdev}(\mathbf{A}\{x, y\}[\nu])$
end for
return $\boldsymbol{\sigma}$

c

Spectral SNR

Require: $\{x, y\} \in \mathbf{B}$
for each $\mathbf{S}_{x,y} \in \mathbf{A}_n$ of n coadditions
  $\boldsymbol{\sigma}_{x,y} \leftarrow \mathrm{stdev}(\mathbf{S}_{x,y})$
end for
return $[\mathrm{mean}(\boldsymbol{\sigma}_{x,y}^{-1}), \mathrm{stdev}(\boldsymbol{\sigma}_{x,y}^{-1})]$

d

Spatial SNR

Require: $\{x, y\} \in \mathbf{B}$
for each $\nu \in \mathbf{A}_n$ of n coadditions
  $\boldsymbol{\sigma}_\nu \leftarrow \mathrm{stdev}(\mathbf{A}_n\{x, y\}[\nu])$
end for
return $[\mathrm{mean}(\boldsymbol{\sigma}_\nu^{-1}), \mathrm{stdev}(\boldsymbol{\sigma}_\nu^{-1})]$

Table S3. Methods of evaluating system spectral and spatial noise, and corresponding SNR for figure 1c(i-iv) respectively. Collection of a hyperspectral dataset (A) with some empty background region (B) is required. The absorbance is calculated at each wavenumber as $A = -\log_{10}(I/I_0)$ by measuring the intensity of the light transflected through the sample (I) and the original intensity of the laser (I_0). The resulting dataset is represented as $A[x, y][\nu]$ where $[x, y]$ are spatial coordinates and $[\nu]$ is the spectral coordinate. (a) The 100% line represents the spectral noise of the system upon detection of 100% of the available light, hence $I = I_0$ and $A = 0$, and is the point spectrum of a single pixel $[x, y]$ located within B, an empty region of the substrate. (b) The spatial noise is calculated from each acquired image per wavenumber. For each spectral band, the standard deviation of the set of pixels $\{x, y\}$ within B is the RMS noise of the system. (c) The spectral SNR is the signal-to-noise ratio calculated from the 100% line. The convention used in fig. 1c(iii-iv) is that the signal is 1 absorbance unit, common for IR spectroscopy, hence the spectral SNR is the inverse of the RMS noise calculated from a single pixel. Calculations are performed at different levels of coadditions (averaged replicate measurements), which are approximately proportional to time and to the square of SNR. Error bars represent one standard deviation from the mean of spectral SNR measurements each calculated from different sets of pixels $\{x, y\} \in B$. (d) Similarly, spatial SNR measurements are calculated from the spatial noise per wavenumber. Error bars are calculated as one standard deviation from the mean of spatial SNR measurements across bands, representing the spread of achievable SNR within the tuning range.

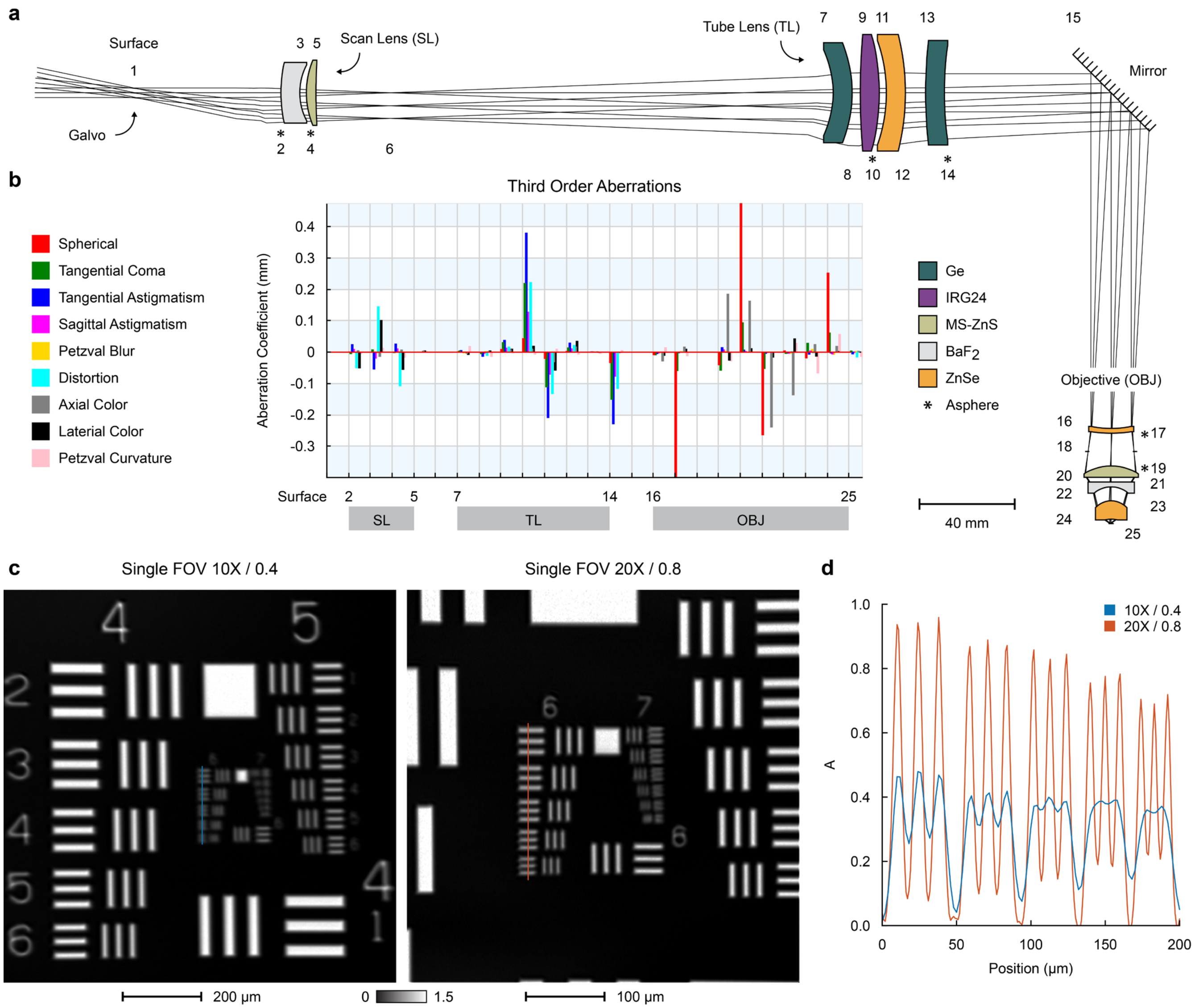

Figure S1. Summary of aberration corrected optical design and performance. (a) Schematic of the full optical system when equipped with the 20X / 0.8 NA objective. (b) Each of these individual assemblies is compensation-free, being fully corrected for aberrations independently of each other. Note that independently for each assembly, the sum of aberration coefficients is ~0. There is potential for simplifying the optical design by sharing the corrective power between the objective and tube lens, albeit with trade-offs associated with backwards-compatibility placed on future designs. This is a strategy employed by some commercial manufacturers of visible microscopy components (e.g. Carl Zeiss Microscopy) but not others (e.g. Olympus Corporation). Here, the xy galvo set is modeled as a single surface, and this simplification causes reduced FOV in the built instrument. Resource permitting, this simplification can be corrected in future designs with a more complicated optical train. (c) Single FOV images of a USAF 1951 chrome on glass resolution test target acquired using both configurations with (d) the indicated line profiles shown. If the intensity of the modulation exceeds ~25% of the maximum, the feature is resolvable per the Rayleigh criterion.

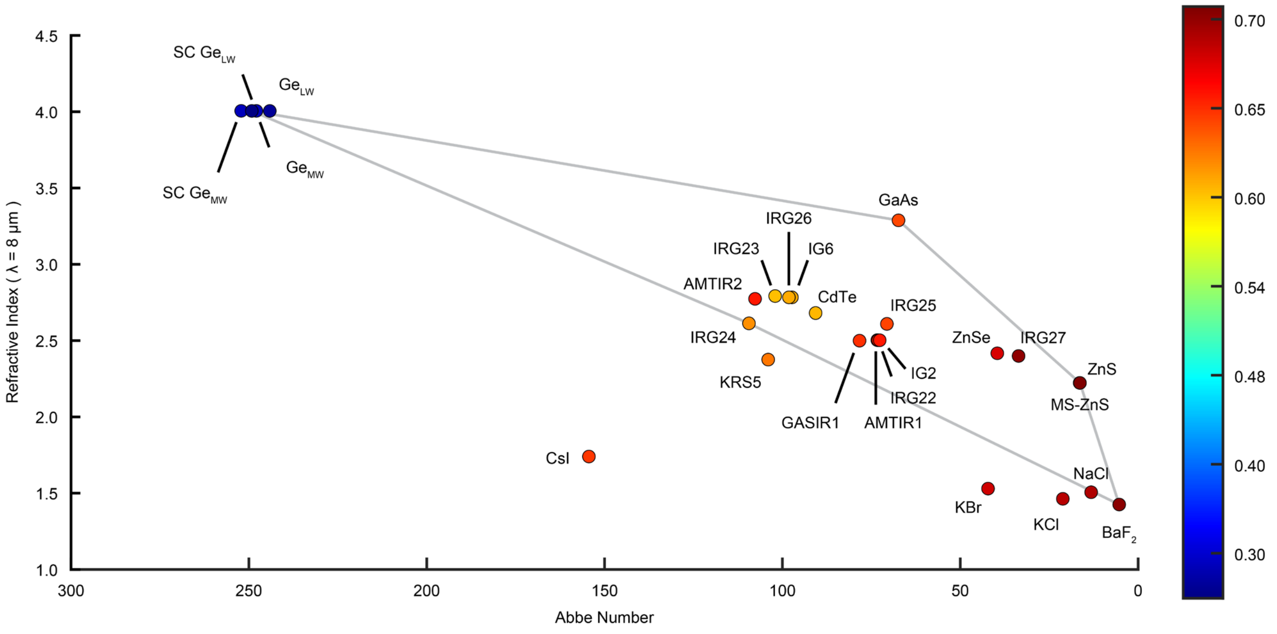

Figure S2. Abbe diagram of the material library filtered for materials that are compatible over the MW/LW IR wavelength range of 5.2-12 µm. Lower Abbe numbers indicate higher chromatic dispersion. The color scale represents the relative partial dispersion of the material. Fictious materials that have properties within the indicated boundary were simulated, allowing a continuous traversal of the Abbe number vs. refractive index vs. partial dispersion search space, until lenses are assigned to real materials by order of similarity. Note the large gaps in the Abbe number and partial dispersion space often causes the optimizer to stall in local maxima/minima, thus many initial design concepts needed to be randomly seeded.

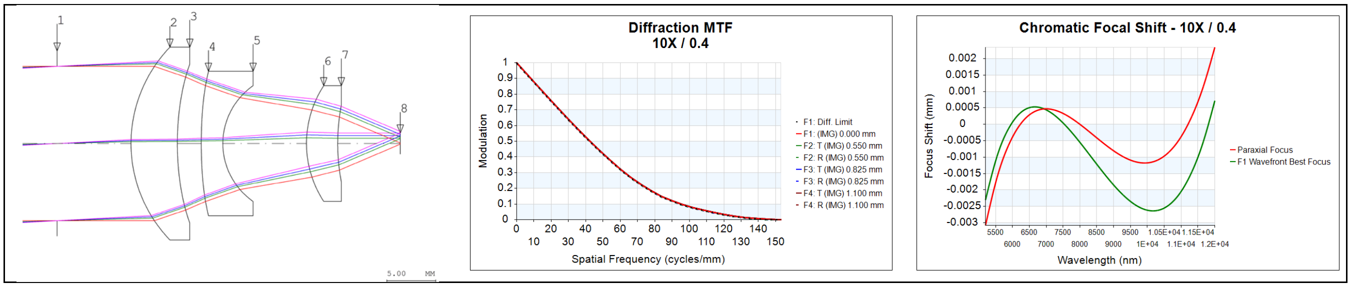

Surface		Radius	Thickness	Material
As	Plane		7.559	
2	Sphere	14.562	4.744	MS-ZnS
3	Asphere	31.076	2.448	
4	Asphere	51.978	2.202	BaF_2
5	Sphere	7.368	8.556	
6	Sphere	11.099	3.191	MS-ZnS
7	Sphere	21.478	6.326	

Aspheric Surface Coefficients		
**Surface**	**3**	**4**
**Conic (κ)**	-1.50013E+00	4.32646E+01
**4th order**	3.46483E-05	2.57100E-05
**6th order**	2.00132E-07	9.91355E-07
**8th order**	-6.35477E-09	-7.54010E-08
**10th order**	1.04914E-10	1.67431E-09
**12th order**	-6.12798E-13	-1.59125E-11

Figure S3. Optical design of the 10X / 0.4 NA microscope objective with each surface type, radius of curvature, surface thickness, and material as indicated. Tabular notation is as follows: surface 1 is represented by the aperture stop (A_S) followed by an air gap of 7.559 mm measured to the vertex of the next surface; surface 2 is the left surface of a MS-ZnS lens with a spherical radius of curvature of 14.562 mm and a substrate thickness of 4.744 mm; surface 3 is the right-side aspherical surface of this lens with a base radius of curvature of 31.076 mm, followed by an air gap of 2.448 mm. This aspheric surface 3 is of the form referenced in the glossary as *, with conic and aspheric coefficients as listed. Subsequent surfaces follow the same convention. The simulated MTF and CFS curves are evaluated at 1250 cm^{-1} and can be compared to experimental results for the overall system in fig. 1d and supp. fig. S9a respectively.

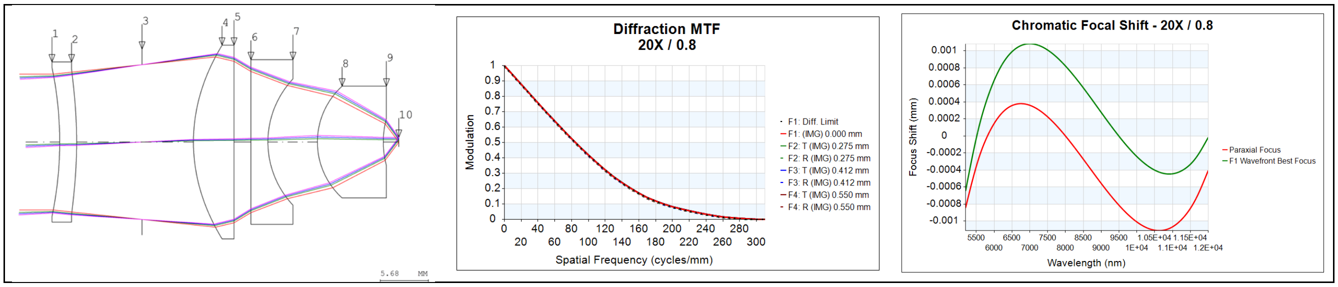

Surface		Radius	Thickness	Material
1	Sphere	-42.819	2.000	
2	Asphere	-54.103	7.718	ZnSe
As	Plane		6.058	
4	Asphere	20.015	4.784	MS-ZnS
5	Plane		2.000	
6	Plane		2.000	BaF_2
7	Sphere	10.853	5.836	
8	Sphere	8.895	7.769	ZnSe
9	Sphere	10.492	1.835	

Aspheric Surface Coefficients		
**Surface**	**2**	**4**
**Conic (κ)**	9.91766E-01	-6.01705E-01
**4th order**	1.31296E-05	3.05056E-06
**6th order**	3.73500E-08	-1.48997E-08
**8th order**	-1.10605E-11	-4.60958E-11
**10th order**	2.09295E-12	9.45898E-15

Figure S4. Optical design of the 20X / 0.8 NA microscope objective following the tabular convention as described in supp. fig. S3. The simulated MTF and CFS curves are evaluated at 1250 cm^{-1} and can be compared to experimental results for the overall system in fig. 1d and supp. fig. S9a respectively.

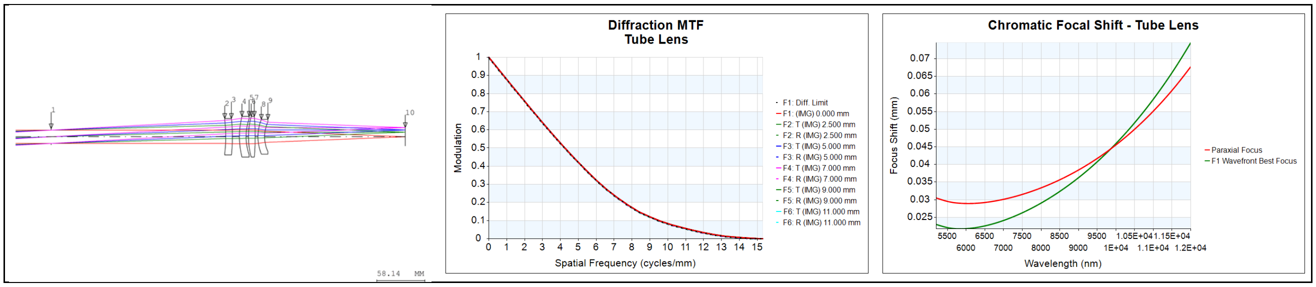

Surface		Radius	Thickness	Material
As	Plane		211.000	
2	Asphere	-167.662	8.000	Ge
3	Sphere	-201.818	8.500	
4	Sphere	100.663	8.000	ZnSe
5	Sphere	65.245	3.000	
6	Asphere	100.672	8.000	IRG24
7	Sphere	-290.832	3.400	
8	Sphere	56.146	8.000	Ge
9	Sphere	42.434	170.000	

Aspheric Surface Coefficients		
**Surface**	**2**	**6**
**Conic (κ)**	-5.11558E-03	5.50328E-03
**4th order**	-5.34824E-07	7.84189E-07
**6th order**	5.38176E-11	-1.86189E-10
**8th order**	-1.00920E-14	5.08952E-14
**10th order**	-1.02227E-18	1.07468E-17

Figure S5. Optical design of the tube lens following the tabular convention as described in supp. fig. S3. The simulated MTF and CFS performance curves were not independently experimentally validated. The overall system performance is shown in fig. 1d and supp. fig. S9a respectively but is dominated by the microscopy objective.

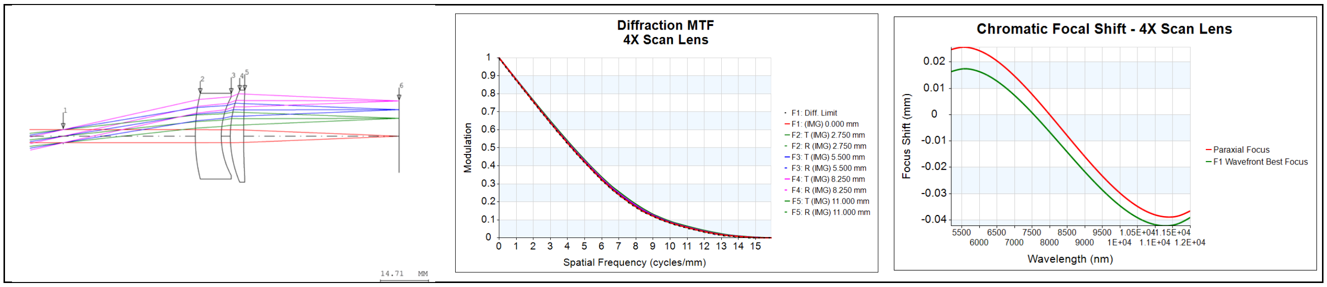

Surface		Radius	Thickness	Material
As	Plane		40.326	
2	Asphere	69.107	7.900	BaF_2
3	Sphere	25.204	2.770	
4	Asphere	35.017	4.060	MS-ZnS
5	Sphere	254.057	47.457	

Aspheric Surface Coefficients		
**Surface**	**2**	**6**
**Conic (κ)**	0.00000E+00	0.00000E+00
**4th order**	9.25519E-06	-2.75031E-06
**6th order**	3.34204E-08	4.69974E-09
**8th order**	-1.15535E-10	-4.81138E-11
**10th order**	-6.15874E-13	2.30915E-13

Figure S6. Optical design of the 4X magnification scan lens following the tabular convention as described in supp. fig. S3. The simulated MTF and CFS performance curves were not independently experimentally validated. The overall system performance is shown in fig. 1d and supp. fig. S9a respectively but is dominated by the microscopy objective.

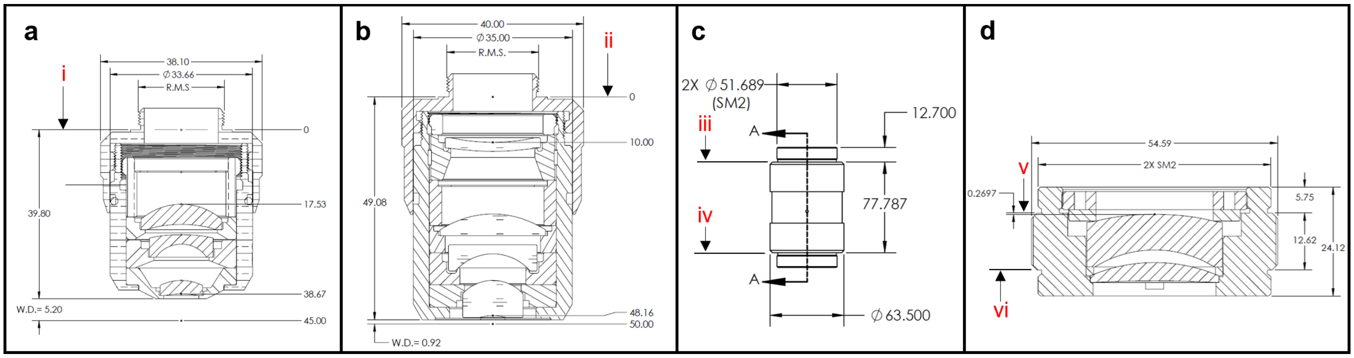

Figure S7. Mechanical drawings of custom refractive compound optics. (a) 10X / 0.4 NA objective. (b) 20X / 0.8 NA objective. (c) Tube Lens. (d) Scan Lens. Reference planes (i) and (ii) are mounted to an objective turret. (a) or (b) is mounted coaxially with (c) and (d) along the optical path no further than 158 mm apart when focused on the sample substrate. This distance is controlled by a motorized focus arm. (c) and (d) are mounted coaxially with reference planes (iv) and (v) 206.35 mm apart. External threading corresponding to (i) and (ii) are RMS class (Royal Microscopical Society) while external threading corresponding to (iii), (iv), (v), and (vi) are SM2 class (Thorlabs). All dimensions are in mm.

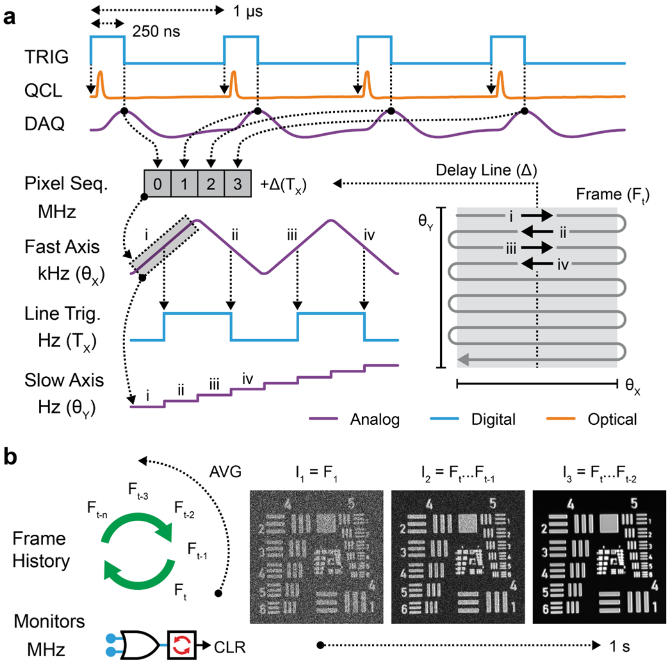

Figure S8. System control schematic of IR-LSM. (a) The laser pulse trigger (TRIG) is a counter output line of the DAQ (PCIe-6361; National Instruments) set with a repetition rate of 1 MHz. This rising edge of this line is used to externally trigger individual QCL pulses. As the DAQ used here has a bandwidth on the order of the repetition rate, far too low to accurately digitize the pulse waveform, we use the falling edge of TRIG to sample the analog input channel connected to the detector, with a delay experimentally determined to be 250 ns. Each analog sample represents the value of a single pixel, measured sequentially at a rate of 1 MHz, and stored (Pixel Seq). The beam is swept using a pair of galvo mirrors (6215H, Cambridge Technology) where the fast axis (θ_X) scans horizontally across the sample with a pseudo-triangular waveform while the slow axis steps vertically. Each row (i-iv) consists of padding (~3.5% per side) where samples are discarded as the fast axis galvo reverses direction. As pixel sizes appropriate for IR wavelengths are large (relative to visible wavelengths), the required sweep speed of the galvo (mechanical degrees per pixel) is proportionally higher for equal pixel rates. When restricted by the maximum bandwidth of the galvo, this results in slower frame rates relative to LSMs designed for visible wavelengths. We rely on bi-directional scanning, eliminating fly-back time, to maximize the duty cycle of the scan (fraction of measurement time out of total time), albeit with the drawback of odd-even line registration. Thus, the zero-crossings (T_X) of the fast axis real-time position output are used to align each row in the frame (F_t) where this tracking is used as feedback to delay (Δ) the pixel sequence, in essence, shifting detector readout relative to actual galvo position. Latencies of each component in this sequence were measured to a precision of 100 ns and included into this system model. (b) The system keeps a running history of all frames acquired in a circular buffer. The coaddition level (n, number of averaged frames) can be adjusted in real-time by

the user with the video feed displaying the average of the previous n frames, without a reduction in apparent frame rate. System monitors detect changes in stage position or wavelength and send a clear (CLR) signal to flush the buffer, thereby avoiding inadvertent coadditions. After CLR, the buffer builds up to n frames, averaging on the fly, as shown above. Not that the level of noise apparent in the images here is simulated for illustrative purposes only. Synchronization of this system is critical when stitching together image tiles for samples that exceed a single FOV, for instance, WSI.

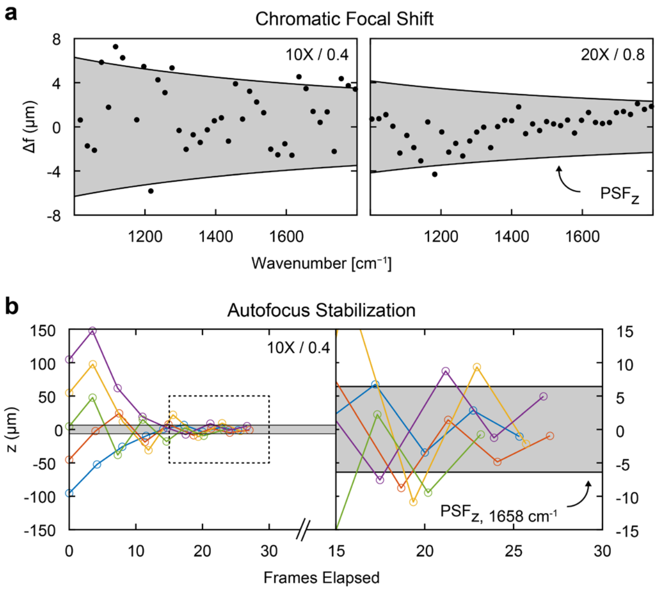

Figure S9. (a) Measured achromatic performance of the full optical system as manufactured, including each microscope objective installed in the microscope with the tube lens and scan lens. (b) The system is also equipped with an autofocus stabilization (AFS) algorithm to correct for long-term system focal drift. Each color indexed trace indicates the z-traversal beginning at different offsets from the true focus at z = 0 µm. This software based AFS assumes that there exists a unimodal distribution at which the maximum focus occurs at the peak of the distribution. AFS consists of two aims: estimation of the best focus position (focus controller), and estimation of the focal quality of the current image (score controller). First, a Fibonacci search algorithm was implemented due to its robustness against noise. Meanwhile, a parallel process grabs image frames from the data stream and calculates a focal score by noise reduction preprocessing and segmentation of the frame into regions of interest targeted by the Features from the Accelerated Segment Test (FAST). The average standard deviation of each of these segments is the calculated score. This process continues until all Fibonacci values in the sequence have been exhausted (no focus found) or if the algorithm has converged (best focus found).
