# Supplementary material for: Infrared spectroscopic laser scanning confocal microscopy for whole-slide chemical imaging
Source: Nat Commun. 2023 Aug 25;14:5215. doi: 10.1038/s41467-023-40740-w (PMC10457288; doi:10.1038/s41467-023-40740-w)
Supplement: Supplementary file 3 — Reporting Summary [file 41467_2023_40740_MOESM3_ESM.pdf]

nature portfolio

Corresponding author(s): Rohit Bhargava

Last updated by author(s): 7/4/2023

# Reporting Summary

Nature Portfolio wishes to improve the reproducibility of the work that we publish. This form provides structure for consistency and transparency in reporting. For further information on Nature Portfolio policies, see our Editorial Policies and the Editorial Policy Checklist.

## Statistics

For all statistical analyses, confirm that the following items are present in the figure legend, table legend, main text, or Methods section.

| n/a | Confirmed | |
|---|---|---|
| ☐ | ☒ | The exact sample size (*n*) for each experimental group/condition, given as a discrete number and unit of measurement |
| ☒ | ☐ | A statement on whether measurements were taken from distinct samples or whether the same sample was measured repeatedly |
| ☒ | ☐ | The statistical test(s) used AND whether they are one- or two-sided<br>*Only common tests should be described solely by name; describe more complex techniques in the Methods section.* |
| ☒ | ☐ | A description of all covariates tested |
| ☒ | ☐ | A description of any assumptions or corrections, such as tests of normality and adjustment for multiple comparisons |
| ☐ | ☒ | A full description of the statistical parameters including central tendency (e.g. means) or other basic estimates (e.g. regression coefficient) AND variation (e.g. standard deviation) or associated estimates of uncertainty (e.g. confidence intervals) |
| ☒ | ☐ | For null hypothesis testing, the test statistic (e.g. *F*, *t*, *r*) with confidence intervals, effect sizes, degrees of freedom and *P* value noted<br>*Give P values as exact values whenever suitable.* |
| ☒ | ☐ | For Bayesian analysis, information on the choice of priors and Markov chain Monte Carlo settings |
| ☒ | ☐ | For hierarchical and complex designs, identification of the appropriate level for tests and full reporting of outcomes |
| ☒ | ☐ | Estimates of effect sizes (e.g. Cohen's *d*, Pearson's *r*), indicating how they were calculated |

*Our web collection on statistics for biologists contains articles on many of the points above.*

## Software and code

Policy information about availability of computer code

| | |
|---|---|
| Data collection | Data collection software (C# .NET Framework 4.8) is available at [https://github.com/chemimage/Cisl.Qclm.Public]. Users will need to contact the authors regarding specific hardware configuration requirements and instructions. No quick setup installer is available. The requestor is responsible for procuring all necessary licenses for 3rd party libraries used, listed in the requirements. |
| Data analysis | MATLAB (R2021a) scripts for processing the collected is available at [https://github.com/chemimage/Cisl.Qclm.Public] |

For manuscripts utilizing custom algorithms or software that are central to the research but not yet described in published literature, software must be made available to editors and reviewers. We strongly encourage code deposition in a community repository (e.g. GitHub). See the Nature Portfolio guidelines for submitting code & software for further information.

## Data

Policy information about availability of data

All manuscripts must include a data availability statement. This statement should provide the following information, where applicable:

- Accession codes, unique identifiers, or web links for publicly available datasets
- A description of any restrictions on data availability
- For clinical datasets or third party data, please ensure that the statement adheres to our policy

The datasets acquired in this study are large (100s GB) so we did not upload them to a public repository. Please refer to the "Data Availability" section in the manuscript: The design files and data sets generated in this study necessary to interpret, verify and extend the research in the article are available from the

corresponding author upon request due to their large file size. Requests for proprietary databases must be directed to the owner, for instance, the Code V material database from Synopsys (Mountain View, CA, USA). Design parameters published in the supplementary files are not intended for manufacturing. The authors recommend the published design be considered an initial guideline and adapted according to the requestor's specific needs, budget, and risk tolerance, as well as the preferences and capabilities of the selected manufacturing partner(s).

## Research involving human participants, their data, or biological material

Policy information about studies with human participants or human data. See also policy information about sex, gender (identity/presentation), and sexual orientation and race, ethnicity and racism.

| | |
|---|---|
| Reporting on sex and gender | All samples used were archival tissue samples and de-identified. The prostate tissue was obtained from a male patient. We do not have information regarding the sex and gender of the patient from which the colon tissue sample was obtained. |
| Reporting on race, ethnicity, or other socially relevant groupings | All samples used were archival tissue samples and de-identified. No information regarding race or ethnicity is available. |
| Population characteristics | All samples used were archival tissue samples and de-identified. No information regarding population is available. |
| Recruitment | All samples used were archival tissue samples and de-identified. No patient recruitment was conducted in this study. |
| Ethics oversight | The use of de-identified tissues was reviewed and approved under Institutional Review Board approval #06684. |

Note that full information on the approval of the study protocol must also be provided in the manuscript.

# Field-specific reporting

Please select the one below that is the best fit for your research. If you are not sure, read the appropriate sections before making your selection.

☒ Life sciences ☐ Behavioural & social sciences ☐ Ecological, evolutionary & environmental sciences

For a reference copy of the document with all sections, see nature.com/documents/nr-reporting-summary-flat.pdf

# Life sciences study design

All studies must disclose on these points even when the disclosure is negative.

| | |
|---|---|
| Sample size | Representative experiments in this manuscript demonstrate the utility of IR-LSM. The reproducibility of the instrument to collect substantively similar data is optimized over years of operation and finally examined in fig. 1 using synthetic ISO standardized targets and methods designed for evaluating the performance of imaging systems. These metrics are reported as noise and apply towards the demonstrative biological samples. The observed variances of the biological measurements are consistent within the noise limit. Thus, sample size calculations are not applicable. 2-4 of each stage of zebrafish embryo, 1 adult zebrafish, 5 frozen prostate tissue sections, and 100 FFPE colon sections were imaged. |
| Data exclusions | No final version data sets were excluded from the statistical analyses. Data sets collected during the course of instrument development from pre-release versions of IR-LSM are not reported. More samples than reported were prepared. We selected which samples to report based on which remained the most intact following the sectioning procedure. Of these samples, we selected which regions to highlight based on morphological interest. Due to the limits of this manuscript, and the intended goal of showing demonstrative images highlighting the performance of IR-LSM, we are not able to show all data recorded. The quality of the data obtained from all samples was substantively equal and consistent within the noise limit. |
| Replication | The imaging capabilities of the microscope were demonstrated across biological samples in fig. 2 & 3 to be consistent within the noise limits characterized in fig. 1. The performance of the DL model described in fig. 4 was replicated by validation on 3 regions excluded from the training set. Three regions were used for validation to maximize the regions used for training (N-3) where N is the total number of regions we were able to obtain across 8 patient biopsies. Sample size was chosen based on the number of these patient biopsies we were able to obtain for a proof-of-concept study. |
| Randomization | As the purpose of the experiments conducted was to demonstrate the imaging performance of the microscope, not biological discovery, this is not applicable. In fig. 1, we characterize the reproducibility of IR-LSM to collect substantively similar data repeatedly and report this metric as noise. In figs. 2 & 3, we demonstrate the bioimaging performance of IR-LSM using a specific animal and a specific human biological sample, not to perform experiments on these samples. In fig. 4, we show that the data quality allows a DL network to segment tissue pathologically; here, the 3 regions were randomly selected for validation and excluded from the training set. |
| Blinding | Blinding is not relevant to fig. 1 as all statistics performed was for the purpose of minimizing noise. Blinding is not relevant to figs. 2 & 3 as these figures demonstrate the bioimaging performance of IR-LSM rather than show biological discovery. For fig. 4, the development of the DL model was not performed by a board-certified pathologist, hence the true pathology of the regions excluded from the training set and used for validation was not known prior to the model's output being evaluated and approved by a board-certified pathologist. |

# Reporting for specific materials, systems and methods

We require information from authors about some types of materials, experimental systems and methods used in many studies. Here, indicate whether each material, system or method listed is relevant to your study. If you are not sure if a list item applies to your research, read the appropriate section before selecting a response.

## Materials & experimental systems

| n/a | Involved in the study |
| --- | --- |
| ☒ | ☐ Antibodies |
| ☒ | ☐ Eukaryotic cell lines |
| ☒ | ☐ Palaeontology and archaeology |
| ☐ | ☒ Animals and other organisms |
| ☒ | ☐ Clinical data |
| ☒ | ☐ Dual use research of concern |
| ☒ | ☐ Plants |

## Methods

| n/a | Involved in the study |
| --- | --- |
| ☒ | ☐ ChIP-seq |
| ☒ | ☐ Flow cytometry |
| ☒ | ☐ MRI-based neuroimaging |

# Animals and other research organisms

Policy information about studies involving animals; ARRIVE guidelines recommended for reporting animal research, and Sex and Gender in Research

| | |
| --- | --- |
| Laboratory animals | Wild type zebrafish (danio rerio) were studied at 28, 52, 76, 100, and 124 hours and ~1.5 years. |
| Wild animals | The study did not involve wild animals. |
| Reporting on sex | Sex was not known for the zebrafish embryo samples and was not considered prior to imaging of the mature zebrafish. |
| Field-collected samples | No field samples were collected. |
| Ethics oversight | The University of Illinois Institutional Animal Care and Use Committee (IACUC) approved the study under protocol #22160. |

Note that full information on the approval of the study protocol must also be provided in the manuscript.
